# The Influence of Oxidative Stress on Serum Albumin Structure as a Carrier of Selected Diazaphenothiazine with Potential Anticancer Activity

**DOI:** 10.3390/ph14030285

**Published:** 2021-03-23

**Authors:** Małgorzata Maciążek-Jurczyk, Beata Morak-Młodawska, Małgorzata Jeleń, Wiktoria Kopeć, Agnieszka Szkudlarek, Aleksandra Owczarzy, Karolina Kulig, Wojciech Rogóż, Jadwiga Pożycka

**Affiliations:** 1 Department of Physical Pharmacy, Faculty of Pharmaceutical Sciences in Sosnowiec, Medical University of Silesia in Katowice, 40-055 Katowice, Poland; kopec.wa@gmail.com (W.K.); aszkudlarek@sum.edu.pl (A.S.); aowczarzy@sum.edu.pl (A.O.); kkulig@sum.edu.pl (K.K.); wrogoz@sum.edu.pl (W.R.); jpozycka@sum.edu.pl (J.P.); 2 Department of Organic Chemistry, Faculty of Pharmaceutical Sciences in Sosnowiec, Medical University of Silesia in Katowice, 40-055 Katowice, Poland; bmorak@sum.edu.pl (B.M.-M.); manowak@sum.edu.pl (M.J.)

**Keywords:** human serum albumin, oxidation, spectroscopic methods, 10*H*-3,6-diazaphenothiazine

## Abstract

Albumin is one of the most important proteins in human blood. Among its multiple functions, drug binding is crucial in terms of drug distribution in human body. This protein undergoes many modifications that are certain to influence protein activity and affect its structure. One such reaction is albumin oxidation. Chloramine T is a strong oxidant. Solutions of human serum albumin, both non-modified and modified by chloramine T, were examined with the use of fluorescence, absorption and circular dichroism (CD) spectroscopy. 10*H*-3,6-diazaphenothiazine (DAPT) has anticancer activity and it has been studied for the first time in terms of binding with human serum albumin—its potential as a transporting protein. Using fluorescence spectroscopy, in the presence of dansylated amino acids, dansyl-l-glutamine (dGlu), dansyl-l-proline (dPro), DAPT binding with two main albumin sites—in subdomain IIA and IIIA—has been evaluated. Based on the conducted data, in order to measure the stability of DAPT complexes with human (HSA) and oxidized (oHSA) serum albumin, association constant (K_a_) for ligand-HSA and ligand-oHSA complexes were calculated. It has been presumed that oxidation is not an important issue in terms of 10*H*-3,6-diazaphenothiazine binding to albumin. It means that the distribution of this substance is similar regardless of changes in albumin structure caused by oxidation, natural occurring in the organism.

## 1. Introduction

Human serum albumin (HSA) is the most important protein in plasma [1]. HSA plays various functions in the human body. It is 70% responsible for maintaining oncotic pressure [2]. Albumin has the ability to bind both exo- and endogenous substances and is an important element in the distribution of drugs in the human body. Binding to albumin may increase the half-life of drugs, reduce drug toxicity, increase solubility and protect against oxidation [3,4]. Protein properties allow for its numerous applications in medicine. It can be used, for example, in skin burns, hemorrhagic shock, ascites caused by cirrhosis of the liver and hypoalbuminemia [4,5]. This protein can be used in the stabilization of vaccines or other therapeutic preparations [4]. HSA is also an effective marker of diseases, including cancer, ischemia and rheumatoid arthritis [5]. Albumin can be used to produce nanoparticles [4] and biomaterials for implantation and surgical patches for closing wounds [5]. 

HSA consists of a single polypeptide chain of 585 amino acids (66.5 kDa) [6]. It mainly contains one tryptophanyl residue at position 214 (Trp-214), 17 tyrosyl residues, 17 disulfide bridges, the only one free cysteinyl residue (Cys-34) [7]. The HSA secondary structure is constituted by 67% α-helix, 23% stretched chain, 10% β-sheets and 35 bends, twists and long polypeptide fragments [8,9]. The tertiary structure of HSA is heart-shaped. It is formed by 8 α-helices and contains three similar domains I, II and III. Each domain is divided into two subdomains, A and B [7]. By the use of fluorescent probes, Sudlow et al. have classified the high-affinity binding sites for drugs—site I and II, called warfarin or phenylbutazone and benzodiazepine or ketoprofen binding sites, respectively [8]. Both site I and site II are located in the hydrophobic cavities of protein, subdomain IIA and IIIA, respectively. Subdomain IIA has a form of pocket in the center of which tryptophanyl (Trp-214) residue is located. It binds dicarboxylic acids and large heterocyclic negatively charged molecules in its center, such as acenocoumarol, azaproprazone, and amantadine. The binding of drugs in subdomain IIA is dominated by hydrophobic reactions. Yamasaki et al. proposed novel nomenclatures for binding site I: regions Ia, Ib and Ic [10], where phenylbutazone binding site corresponds to the region Ia and Ib. IIIA subdomain is a binding place where aliphatic ligands or aromatic carboxylic acids bind to the negatively charged acid group at the end of the molecule, away from its hydrophobic center. There are located that is, three tyrosyl residues Tyr-401, Tyr-411, Tyr-497. In the IIIA subdomain, drug bonds are accompanied by hydrophobic and electrostatic interactions as well as hydrogen bonds [4,8]. The L-shaped binding pocket in subdomain IB has recently been identified as another binding site for compounds with slightly different properties [4]. It contains residues Arg-117, Arg-186, five tyrosyl residues, and can form hydrogen bonds [4,8]. 

Albumin undergoes many in vivo reactions that may affect its structure and thus its interactions with other substances. One of such in vivo reactions is oxidation, which is intensified in the case of diseases, for example, cancers [11]. Cancer cells produce much more reactive oxygen species (ROS) than unchanged cells [12,13]. Then the concentration of free radicals increases, which generates oxidative stress. ROS have a destructive effect on proteins, lipids of cell membranes and nucleic acids [14]. The influence of free radicals on the structure of proteins is manifested in the modification of amino acid residues and prosthetic groups in complex proteins. The direct effect of protein oxidation is the breaking of the polypeptide chain and the formation of cross-links between one or more polypeptide chains. These changes result in an increase or loss of biological activity [15]. Tyrosines and tryptophan residue that are presented in the main albumin binding sites are highly sensitive to oxidation [16]. HSA is a mixture of mercaptalbumin (HMA, reduced form) containing one free sulfhydryl group in Cys-34 (potentially important target for oxidative stress) and nonmercaptalbumin (HNA, oxidized form) which is comprised of at least three types of molecules—mixed disulfide with cysteine and glutathione and products oxidized HNA to a greater extent than mixed disulfide [15].

**Figure 1:**
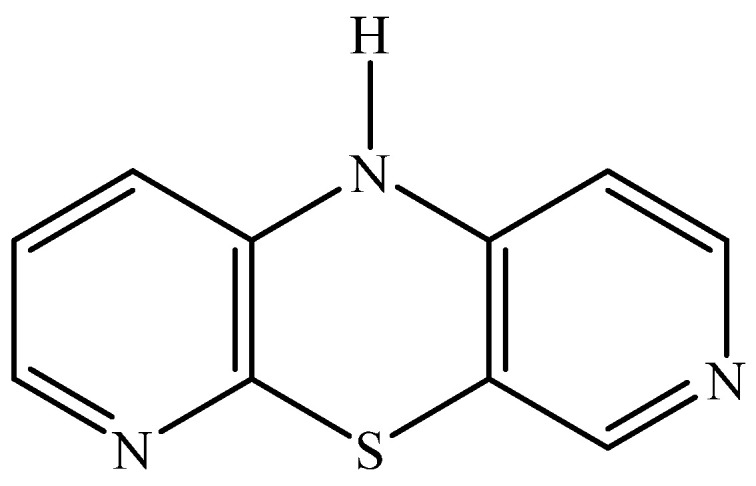
Structural formula of 10*H*-3,6-diazaphenothiazine (DAPT).

Cancer diseases are characterized by uncontrolled processes of cell proliferation and growth, which affect the homeostasis of the organism. These civilization diseases are the result of genetic and epigenetic changes in the body. Recently, an increase in cancer incidence has been observed, which has led to the focus of science on the synthesis of substances with anticancer properties. Tricyclic phenothiazines have chemical and biological properties that have made their use in medicine possible. Compounds from this group show antitussive, antiemetic, antihistamine and neuroleptic properties. The structural modifications of the phenothiazines consist mainly in the substitution of a nitrogen atom in the ring or the replacement of benzene rings with homoaromatic and heteroaromatic rings in order to obtain benzophenothiazines or azaphenothiazines [17,18]. These changes aim to increase their potency and activity. One of the tricyclic azaphenothiazines is 10*H*-3,6-diazaphenothiazine (DAPT) (Figure 1).

10*H*-3,6-diazophenothiazine (DAPT) has promising anticancer properties and low cytotoxicity. It has been obtained by cyclization of 3′-nitro-2,4′-dipyridyl sulfide and by reaction of sodium 3-amino-2-pyridinethiolate with 4-chloro-3-nitropyridine. This compound is 10 times more active against the SNB-19 glioblastoma, C-32 melanoma and MCF-7 breast cancer cell lines than the reference drug cis-platin. Half of the maximum inhibitory concentration is in the range of IC_50_ = 0.46–0.72 µg·mL^−1^. Analysis of mechanism of anticancer action showed, that 10*H*-3,6-diazaphenothiazine (DAPT) exhibits antiproliferative properties by activating the p53 protein pathway in cancer cells, which causes the cell cycle to stop and makes the cancer cell go to sleep. The *BAX/BCL-2* gene expression ratio suggests activation of the mitochondrial apoptosis pathway in SNB-19 and MCF-7 cells. Moreover, DAPT is non-toxic to healthy human fibroblasts (HFF-1) [19]. Further studies confirmed the promising, high antiproliferative activity of DAPT in relation to ovarian cancer A2780 (IC_50_ = 0.62 μM) and significant low toxicity towards normal kidney HEK293 cells and normal heart H9C2 cells. Additionally DAPT has induced both intracellularly and extracellularly apoptosis pathway. It affected the activation of the caspase chain and the regulation on NF-κB and [BIRC6-XIAP] complexes in the analysis of anticancer action [20].

Human serum albumin, as one of the main substances transporting exogenous and endogenous compounds, affects the bioavailability of many drugs. In a living organism, it undergoes modifications that affect its binding sites. One of these processes is oxidation, which may occur during an ongoing cancer process. Protein binding of drugs influences the blood concentration of the drug and therefore its pharmacological effect. The main aim of the study was to evaluate the interaction (binding) of unmodified and oxidized human albumin with 10*H*-3,6-diazaphenothiazine (DAPT). Dansylated amino acids were used to locate the 10*H*-3,6-diazaphenothiazine (DAPT) binding site in macromolecule. 10*H*-3,6-diazaphenothiazine (DAPT) is a newly synthesized substance with potential antitumor activity and these studies are a novel approach to the subject of cancer therapy. 

## 2. Results and Discussion

### 2.1. In Vitro Assessment of Oxidative Albumin

Oxidized albumin (oHSA) is a reliable marker of oxidative stress and oxidative stress has been linked to a number of chronic diseases (i.e. cancer diseases) [21]. Oxidative stress induces a cellular redox imbalance which has been found to be present in various cancer cells compared with normal cells. Several models of oxidation indicate that the changes in structure and activity of oxidized proteins affect the binding properties [22]. Maciążek-Jurczyk et al. wrote that conformational changes in HSA via oxidation result in a decrease in its drug binding capacity and antioxidant activity [23]. The effect of oxidation on ligand binding is dependent on the oxidant used. Oettl and Stauber [24] described the methods of generating oxidized albumin like incubations with ascorbic acid in the presence of oxygen and metal ions (trace amounts or μmol·L^−1^ concentrations added), hydrogen peroxide, chloramine T, glucose, cysteine or homocysteine and NO. In the present study, chloramine T (ChT) was used to oxidize albumin. Since chloramine T generates the formation of hydroxyl radical and chlorine radicals, it can mimic the oxidation reactions taking place in the human organism [15]. 

#### 2.1.1. Absorption Spectra of Albumin 

**Figure 2:**
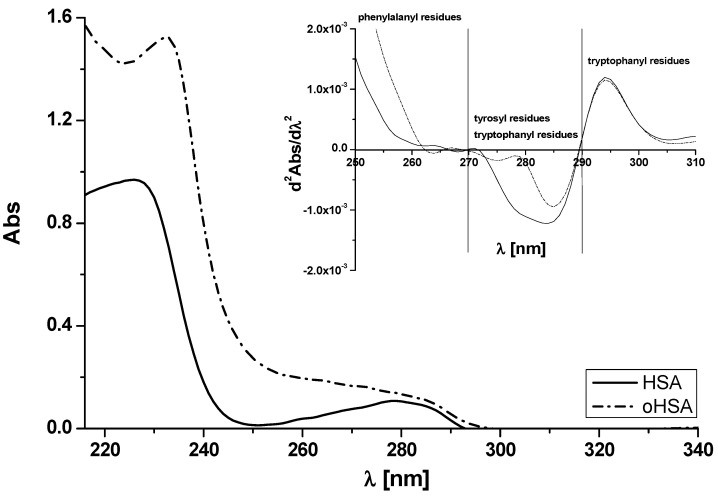
Absorption spectra of unmodified (human serum albumin (HSA)) and oxidized (oxidized human serum albumin (oHSA)) human serum albumin at 5 × 10^−6^ mol·L^−1^ concentration. In the insert second derivative of 5 × 10^−6^ mol·L^−1^ HSA and oHSA absorption spectrum.

The oxidation of individual amino acid residues by chloramine T depends on its concentration [15]. In the present study, according to previously described protocols [11,16], chloramine T (ChT) at 1 × 10^−3^ mol·L^−1^ concentration (ChT:HSA = ChT:oHSA 25:1) has been used in order to oxidize human serum albumin. Figure 2 shows HSA and oHSA absorption spectra ([HSA] = [oHSA] 5 × 10^−6^ mol·L^−1^).

Based on Figure 2 it can be observed that oHSA absorption is higher than HSA. Absorbance values corresponding to the absorption wavelength of amino acid residues located in the main protein binding sites, such as 52 phenylalanine residues located almost in the entire albumin structure, tryptophan residue (Trp-214) located in subdomain IIA and 20 tyrosyl residues located mainly in subdomain IB (Tyrs-140, 148, 150, 156, 157) and IIIA (Tyrs-401, 411, 452, 497) at λ 278 nm are 0.11 and 0.14 for HSA and oHSA, respectively. ChT probably alters human serum albumin structure in the environment of phenylalanine, tyrosyl or/and tryptophanyl residues (A_278 nm_). As Meucci et al. concluded, mild oxidative stress, as elicited by ascorbate, oxygen, and trace metals, affects the binding properties of human serum albumin via purely conformational changes [25]. They incubated HSA at 37 °C with ascorbate system and discussed modifications that is, by the changes (increases) in absorption spectra. According to them, oxidized protein is more heat resistant, less flexible and more rigid than the unmodified one leading to the loss of biological activity. This appears to be of medical relevance, because it can affect drug metabolism and particularly drug tolerance in the elderly or with cancer. It is well known that the absorption spectrum of protein in the range between 250 and 300 nm derives from phenylalanine, tyrosyl or/and tryptophanyl residues and it is necessary to resolve the complex protein absorption spectrum into the individual contributions of these three aromatic amino acids residues. Therefore, absorption spectra second derivative of HSA and oHSA have been registered (Figure 2, insert) in order to show how quickly the absorbance rate changes. The range between 250 and 270 nm spectrum illustrates the rate of change within phenylalanine residues, the range between 270 and 290 nm illustrates changes in tryptophanyl and tyrosyl residues environment and between 290 and 310 nm only in tryptophanyl residues. Based on the similar course of HSA and oHSA absorption spectrum second derivative in the range between 250–300 nm the same concentration of both samples has been confirmed. In the region 250–310 nm significant differences were recorded, the biggest within the tryptophanyl an tyrosyl residues (270–290 nm) while the smallest within only the tryptophanyl residue (290–310 nm). Similar to the previous work [26], it can be concluded that the second derivative of the human serum albumin spectrum has been employed for detecting conformational changes involving the microenvironments of aromatic amino acid residues of oHSA, especially tyrosyl residues. However, literature data prove that the main target of oxidative stress are protein thiols. Oxidation of the –SH group in cysteine plays a critical role in the folding process of protein chains and is thereby important for peptides and proteins function and stability. The 5,5′-dithiobis(2-nitrobenzoic acid) reagent (DTNB) has been developed by Ellman as a sulfhydryl reagent. DTNB is a sensitive tool for the assay of thiol groups not only in tissues and body fluids but also in proteins [27]. DTNB reacts with thiol –SH residues forming disulfide bonds and strongly absorbs at λ 412 nm. Based on the method described previously [11,16], absorption spectra of HSA and oHSA in complex with Ellman’s reagent have been made and molar concentration of free sulfhydryl groups using Equation (1) have been calculated and equals to 1.76 × 10^−6^ mol·L^−1^ for HSA and, upon influence of oxidation (oHSA), decreased to 0.18 × 10^−6^ mol·L^−1^. Based on Equation (2), the percentage of free sulfhydryl groups [SH] in unmodified (4.40%) and oxidized (0.44%) albumin, which determines the amount of free sulfhydryl groups per 100 molecules of albumin has been obtained. Although presented above data (second derivative absorption spectra) point to the slight changes in oxidized protein IIA, IB or/and IIIA subdomains, these quantitative data confirms that chloramine T destabilizes conformation of serum protein and causes [SH]% values reduction. These results are consistent with Tawfik who briefly described a sulfhydryl groups modification with DTNB [28]. Cys-34 is a single thiol which accounts for ~80% of reduced thiols in human plasma. Cys-34 has a utility as biomarker of oxidative reactions and is used for the characterization and quantification of the oxidative modifications in albumin [14,15]. Provided by X-ray diffraction the HSA three-dimensional structure confirms that Cys-34 is located in a hydrophobic crevice of 9.5–10 Å (close to subdomain IB) with no other adjacent thiol, surrounded by side chains of Pro-35 and Val-77. In addition, by the imidazole ring from His-39, the carboxylate group of Asp-38 and also the hydroxyl group of Tyr-84 (subdomain IA) [29,30,31]. Because subdomain IB is the third major binding region of human albumin, drug binding with oxidized form of albumin, due to negative effect on the binding site, should be taken into account. Kaneko et al. have reported that subdomain IA maybe also the drugs’ binding site that is, the site of the allosteric human serum albumin binding of ketoprofen at pH 8.2 [32]. Taking it into account, the crucial role of oxidation in albumin binding properties has been confirmed.

#### 2.1.2. Emission and Excitation Spectra of Albumin 

**Figure 3:**
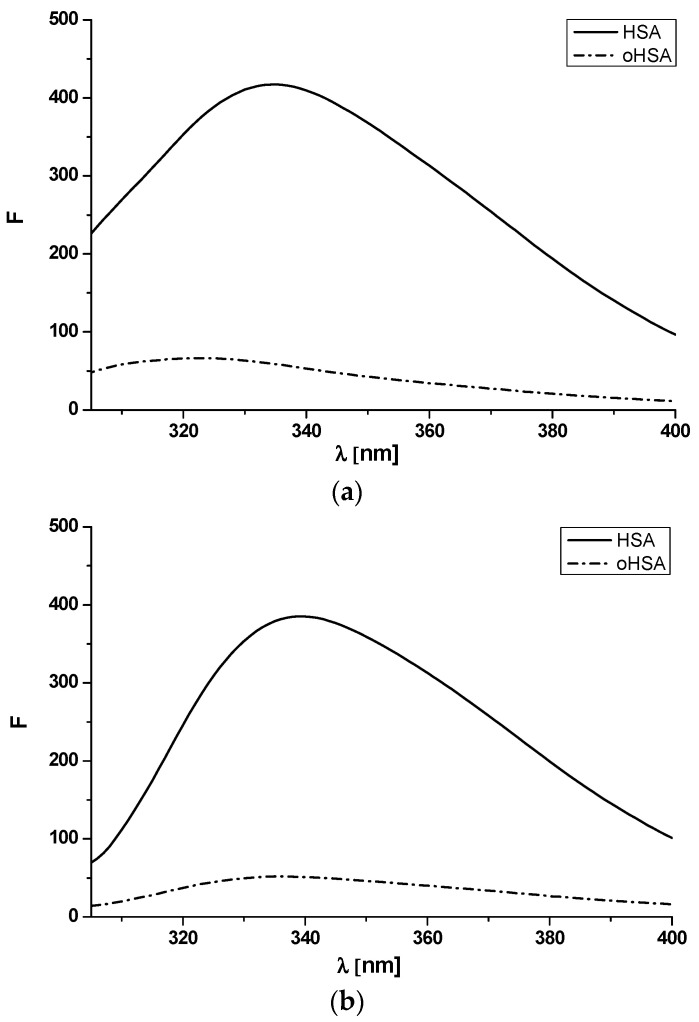
Emission fluorescence spectra of unmodified (HSA) and oxidized (oHSA) human serum albumin at 5 × 10^−6^ mol·L^−1^ concentration excited at (**a**) λ_ex_ 275 nm and (**b**) λ_ex_ 295 nm.

It is well known that the excitation at λ_ex_ 275 nm wavelength results in fluorescence emission derived from tyrosyl and tryptophanyl residues, whereas the 295 nm excitation wavelength causes fluorescence emission of tryptophanyl residue. Emission fluorescence spectra of HSA and oHSA at 5 × 10^−6^ mol·L^−1^ concentration and excitation wavelengths λ_ex_ 275 nm and λ_ex_ 295 nm has been shown in Figure 3.

As has been shown in Figure 3, the spectrum of oxidized oHSA is less intense than the spectrum of HSA and shifts in the shortwave direction (λ_ex_ 275 nm). To confirm the short-term shift of the fluorescence band, the spectral parameter A has been calculated. Spectral parameter A (A = F_365 nm_/F_320 nm_) was used because of its sensitivity to small changes in the position of maximum fluorescence wavelength (λ_max_) [33]. Moreover the changes in HSA conformation under an influence of oxidation reagent have been studied based on full width at half maximum (FWHM) values (Table 1). 

A decrease in albumin fluorescence intensity, parameter A and FWHM values has been attributed to tertiary structure changes. This phenomenon, probably due to the decrease in exposure of oHSA tryptophanyl and tyrosyl residues to the solvent, indicates an increase in the hydrophobicity of the fluorophore environment—tyrosyl and especially tryptophanyl residue which is very sensitive to the nature of the immediate surroundings. A reduction in tryptophanyl residue intensity accompanied by as light blue shift has been reported by Anraku et al. [14] and Maciążek-Jurczyk et al. [16]. They suggested that the observed drop in albumin fluorescence intensity could be caused by the exposure of tryptophanyl residue to the solvent and/or quenching of tryptophanyl residue intensity with an increase in the number of the rest of phenylalanines, histidines, and disulfides in the proximity of tryptophanyl residue. Due to the changes manifesting the conformational variations of the protein under the influence of oxidation we can concluded that not only subdomain IIA where tryptophanyl residue (Trp-214) is located, but also other subdomains of protein containing tyrosyl residues (Tyr) have been modified.

As we can see, human albumin fluorophores are sensitive to changes in their area, and therefore changes in the structure of albumin. These changes are noticeable in the fluorescence intensity (decrease) of tyrosyl and tryptophanyl residues. In order to track changes in the fluorophore microenvironment, an analysis of the REES (Red Edge Excitation Shift) phenomenon should be performed [34]. The REES application is a powerful tool to monitor the organization and dynamics of a variety proteins fluorophores environment [35]. The REES effect relies on shifting the emission maximum towards higher wavelengths and is caused by the excitation wavelength gradually shifted to the red edge of the absorption band [36]. In order to study this effect, both HSA and oHSA were excited at λ_ex_ 290 nm, λ_ex_ 295 nm, λ_ex_ 300 nm, λ_ex_ 305 nm wavelengths. Data collected from emission spectra (spectra not shown) have been summarized in Table 2. 

Based on our results, we can conclude that with the increase in the excitation wavelength, a shift of the emission spectrum, 3 nm for unmodified albumin and 12 nm for oxidized albumin, towards longer wavelengths is observed. This phenomenon confirms that tryptophan excited at from λ_ex_ = 290 nm to λ_ex_ = 305 nm shows REES and indicates the localization in environments (subdomain IIA) which are motionally restricted due to slow solvent relaxation. By studying the REES effect, the reduction of mobility in the oxidative protein environment in the vicinity of the fluorophore due to the stiffening of protein matrix can be observed [37]. However Geddes et al. [35] wrote that tryptophans residues in modified by denaturation proteins generally do not exhibit REES due to fast solvent relaxation in the denatured state. Demchenko explained it that the tryptophans residues are exposed to water when denatured and therefore do not offer any restriction to the solvent (water) dipoles around them in the excited state [36]. 

**Figure 4:**
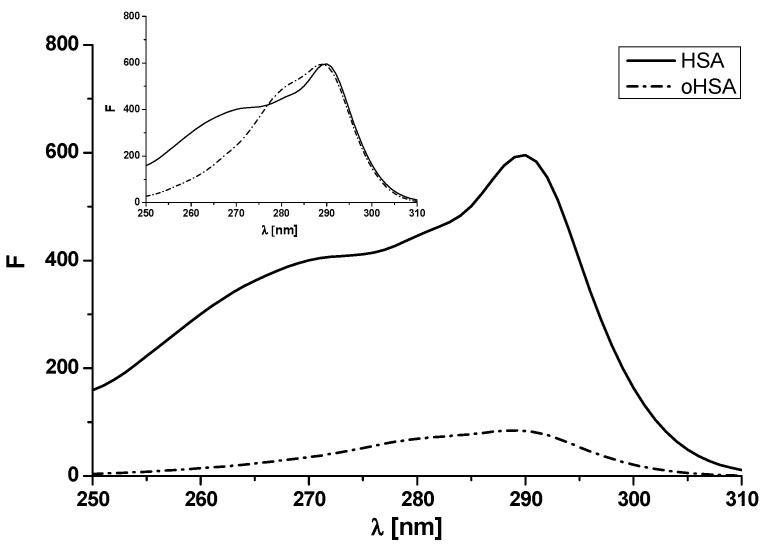
Excitation fluorescence spectra of unmodified (HSA) and oxidized human serum albumin (oHSA) at 5 × 10^−6^ mol·L^–1^ concentration. In the insert normalized excitation fluorescence spectra of oHSA and unmodified HSA.

As proved in the previous chapter, oxidation influences the albumin structural changes, which was shown, that is, by changes in albumin absorption spectra and their second derivatives. In order to confirm this theory, it was also proposed to obtain excitation fluorescence spectra of unmodified (HSA) and oxidized human serum albumin (oHSA) at 5 × 10^−6^ mol·L^−1^ concentration. The samples excited at 250–310 nm, and the emission signal was observed for the constant emission wavelength λ_em_ 340 nm (Figure 4). The oxidized oHSA spectrum has been normalized against unmodified HSA (Figure 4, insert).

Based on the presented data, a decrease in the fluorescence of oxidized versus unmodified albumin was observed. At λ_max_ 290 nm, the fluorescence intensity of HSA was 595.67 while for oHSA was 83.69 (Figure 4). Moreover, apart from changes in fluorescence intensity, the spectrum of oxidized versus unmodified albumin has a changed shape (Figure 4, in the insert). The normalized excitation spectra differ significantly at the shorter wavelengths and with increasing wavelengths, the spectra start to overlap. This phenomenon confirms the fact that oxidized oHSA albumin has a different structure than unmodified HSA. Fluorescence excitation spectroscopy has been utilized by Rokos et al. [37] to study in vivo H_2_O_2_–mediated oxidative stress in the depigmented epidermis of untreated patients with vitiligo. Using human albumin they confirmed that the fluorescence excitation spectrum of HSA in vitro is dominated by the fluorescence of the single tryptophan residue in its sequence at position 214 and the λ_max_ of this spectrum was concentration dependent yielding a maximum at 295 nm (10^−3^ mol·L^−1^) shifting to a maximum at 280 nm at concentrations below 2 × 10^−5^ mol·L^−1^). 

#### 2.1.3. Circular Dichroism (CD) Spectra of Albumin 

**Figure 5:**
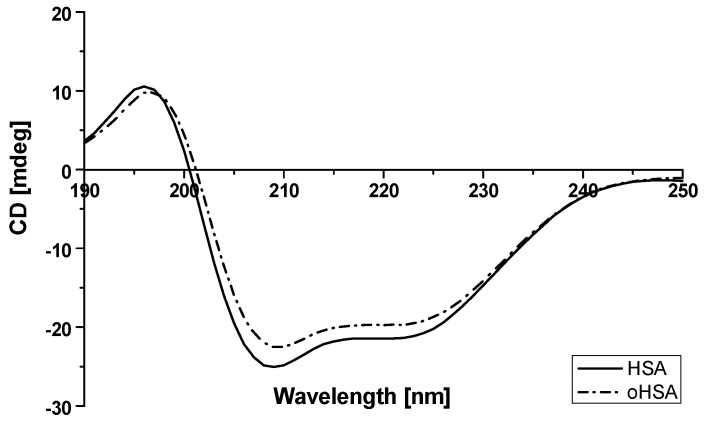
Circular dichroism (CD) spectra of HSA and oHSA at 1 × 10^−6^ mol·L^–1^ concentration.

Circular dichroism (CD) is an absorption spectroscopy method based on the differential absorption of left and right circularly polarized light [38]. Proteins far ultraviolet CD spectrum (far-UV CD) below 250 nm reflects the secondary structure of the protein, that is, the α-helical structure, β-sheet, β-turn and unstructured elements [39]. As it is known, the all α-proteins CD spectrum shows a strong double minimum at 222 nm and 208–210 nm and a stronger maximum at 191–193 nm. All β-proteins usually have a single negative band in the 210–225 nm wavelength range and a stronger single positive band in the 190–200 nm wavelength range. CD is a powerful tool for investigating the structure of proteins even during the natural modifications [40]. It provides signs of protein destabilization (modification) and α-helix reduction [41,42]. CD measurements of HSA and oHSA were performed to assume the oxidative stress effect on their secondary structure and are presented in Figure 5.

The observed CD spectrum of HSA shows double minimum at 209 nm and 223 nm and it proves that HSA is a α-helical protein (Figure 5). Moreover ellipticity (deg) at λ 200 nm and 250 nm was similar, which means that the concentration of HSA and oHSA was the same. An incubation of HSA with chloramine T resulted in an increase in observed ellipticity (mdeg). Based on Equation (3), the mean residue ellipticity [θ_MRW_], both for HSA and oHSA, has been calculated and collected in Table 3. 

For oHSA a θ_MRW_ value is 0.90 times higher than for HSA for the first λ_min_ 209 nm and 0.92 for the second one λ_min_ 223 nm. As was previously mentioned, β-type proteins contain only one negative band around λ_min_ 220 nm and one positive around 195 nm. This slightly stronger increase in the mean residue ellipticity for the 223 nm band may indicate a decrease in the content of α-helical structures and an increase in β-structural elements. Due to the oxidation process, similar to the fibrillation/aggregation process [15], HSA probably changes from the native state characterized by the presence of two minima at 223 and 209 nm to a spectrum with a visible minimum and less visible second one as a consequence of β-sheet structure, absent in unmodified HSA, as Kosa et al. explained in their work [43]. Using the Secondary Structure Estimation program with the Yang’s reference model, a reduction in the α-helical content of protein structure and the formation of β-sheet conformation (the increase in %β-sheet value) have been confirmed. Data have been collected in Table 4.

Based on the presented data we observed changes in the proportion of α-helix and β-sheet structures of HSA after incubation with chloramine T (ChT). These results have been explained by Paris et al. [44] by the reduction of the protein compactness and consequent destabilization of the secondary structure owing to the exposure of hydrophobic sites to the solvent after a probable reduction of S–S bridges. Circular dichroism showed complex results, in which ChT was a strong modifier of the percentages of the α-helical structure of HSA. Monacelli et al. have studied structural alterations of human serum albumin caused by glycative and oxidative stressors by circular dichroism analysis [41]. They used ribose with diethylenetriamine pentacetate (DTPA) and ascorbic acid (AA) in the presence of DTPA combinations and DTPA alone as effective modifiers of secondary protein structure. Similar to our study, Monacelli et al. observed that when the α-helix structure decreased, the β-sheet structure also increased, reflecting the modification of human serum albumin properties. 

### 2.2. HSA Binding Sites Assessment 

Under the influence of oxidation stress many proteins present changes in the secondary and tertiary structure. Protein oxidation is a destructive process leading to the degenerative diseases ranging from cancer and heart disease to diabetes. Human serum albumin is a transporting protein of many exo- and endogenous substances and the changes in albumin binding sites can have a fundamental role in the pharmacological effect. Thus, the study of the oxidation of human serum albumin and its influence on the main binding sites is essential.

#### 2.2.1. Dansyl-l-Glutamine (dGlu) and Dansyl-l-Proline (dPro)—HSA and oHSA Binding

**Figure 6:**
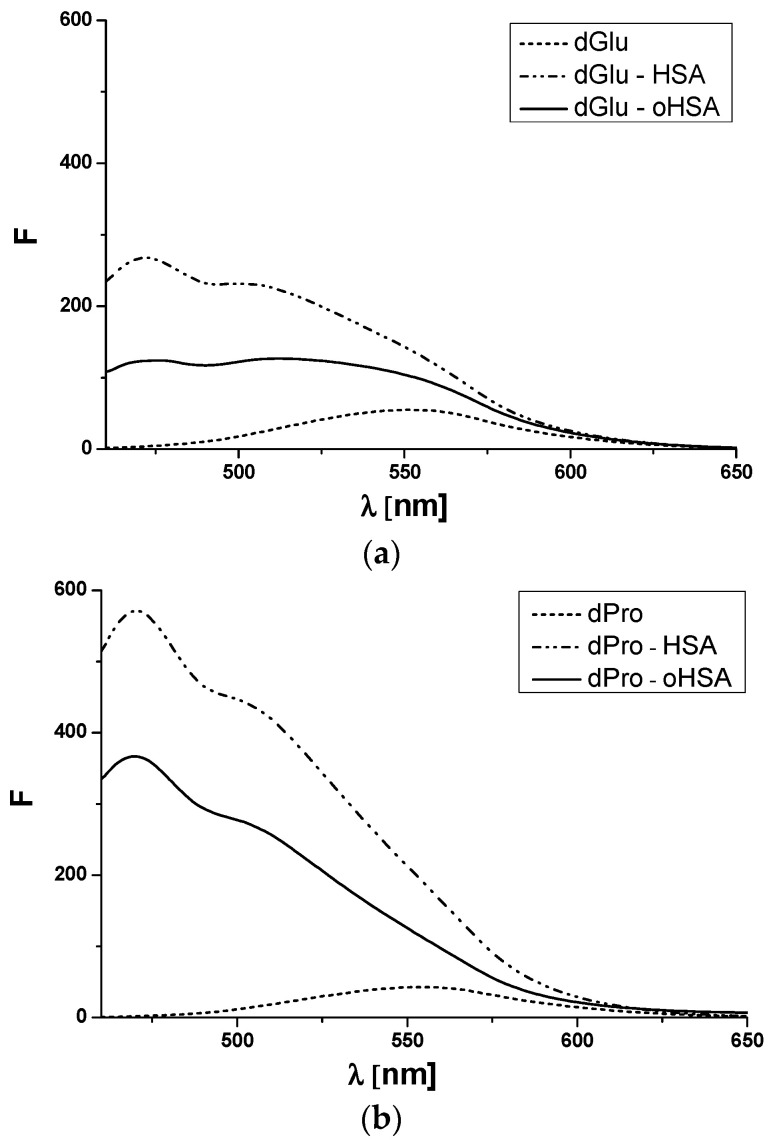
Emission fluorescence spectra of (**a**) dGlu and (**b**) dPro in the presence of HSA and oHSA. [dGlu] = [dPro] 5 × 10^−5^ mol·L^−1^, [HSA] = [oHSA] 1 × 10^−5^ mol·L^−1^; λ_ex_ = 350 nm.

In the HSA structure there are several main drug binding sites called Sudlow’s site I in IIA subdomain, Sudlow’s site II in IIIA subdomain and binding site in IB subdomain [45]. In order to assess the number and location of drug binding sites on the HSA molecule, both oxidized (oHSA) and unmodified (HSA), dansylated amino acids have been studied. Dansylated amino acids have different structures that influence the binding to specific albumin binding sites. Containing hydrophobic side chains, amino acids like dansyl-l-Proline (dPro) bind to Sudlow’s site II (IIIA subdomain), while amino acids with polar side chains or electric charge like dansyl-l-Glutamine (dGlu) bind to Sudlow’s site I (IIA subdomain) [46]. Fluorescence spectra of dPro and dGlu, in the presence of both unmodified and oxidized albumin, confirm amino acids binding in the specific binding sites (Figure 6). 

**Figure 7:**
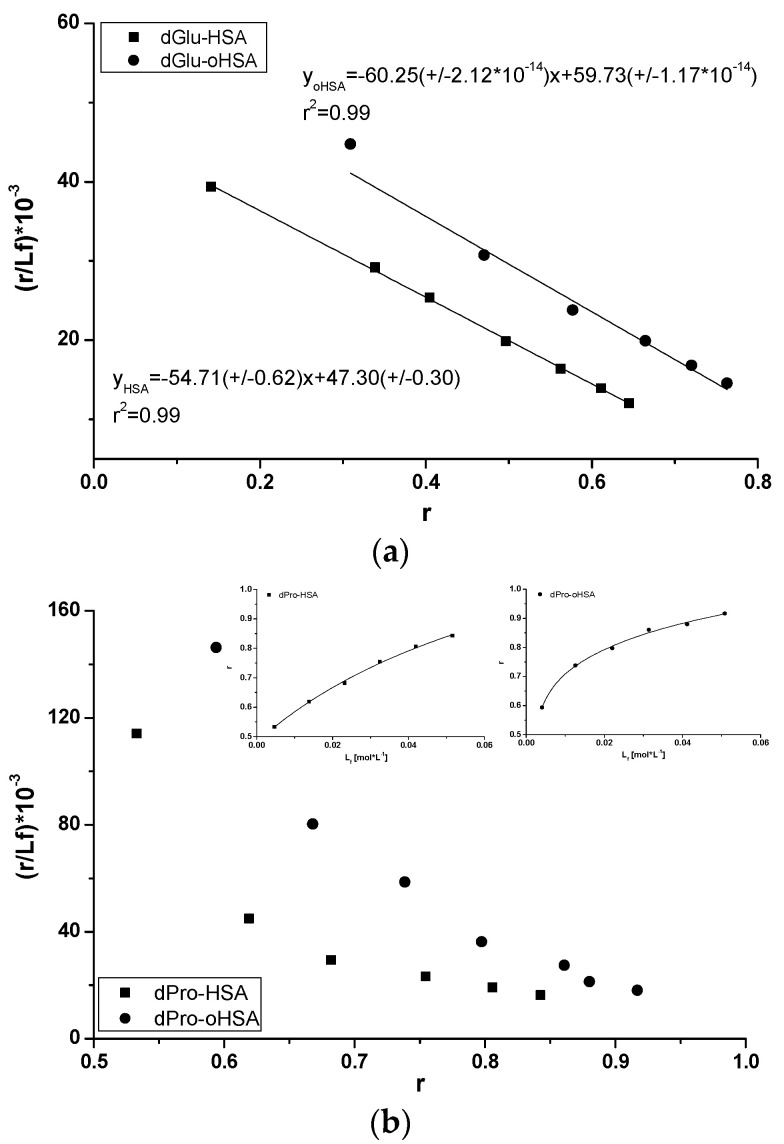
Scatchard curve for (**a**) dGlu-HSA, dGlu-oHSA and (**b**) dPro-HSA, dPro-oHSA systems; λ_ex_ 350 nm. [HSA] = [oHSA] 1 × 10^−5^ mol·L^−1^, [dGlu] 0.5 × 10^−5^ mol·L^−1^–6 × 10^−5^ mol·L^−1^. In the insert the binding isotherms for dPro-HSA and dPro-oHSA systems.

Fluorescent label dGlu binds to the IIA subdomain of HSA molecules while dPro to HSA IIIA subdomain [46,47]. This phenomenon has been confirmed by the analysis of dGlu (Figure 6a) and dPro (Figure 6b) fluorescence spectra. The binding with HSA and oHSA causes a shift towards short wavelengths of dGlu (Δλ 21 nm, Δλ 26 nm) and dPro (Δλ 16 nm) spectra and confirms the location of both probes in albumin hydrophobic regions. Due to the changes in dGlu and dPro fluorescence excited at 350 nm in the presence of HSA and oHSA, dGlu-human serum albumin binding in the IIA subdomain and dPro-human serum albumin binding in the IIIA subdomain have been studied. Using the data collected from the fluorescence spectra of dGlu-HSA and dPro-HSA complexes (data not shown), association constants K_a_ [mol^−1^·L] have been calculated (Figure 7).

As has been calculated, association constants for dGlu-HSA and dGlu-oHSA system equal to K_a_ = 5.47 × 10^4^ ± 0.62 × 10^4^ mol·L^−1^ (n = 0.86 ± 0.01) and 6.02 × 10^4^ ± 2.12 × 10^−10^ mol·L^−1^ (n = 0.99 ± 0.01), respectively (Figure 7a). The slightly higher value of dGlu association constant in the presence of oHSA compared to HSA indicates that the oxidation process changed the IIA subdomain of albumin, however, the change in the K_a_ value is small and points to non-significant modifications. Similarly as in our previous study [26], the Scatchard plots obtained for both dPro-HSA and dPro-oHSA systems are not straight lines (Figure 7b). It probably means that there are more than one binding site for dPro in HSA and oHSA subdomains. The association constants K_a_ [mol·L^−1^] obtained for two binding sites were determined using the non-linear regression method (Figure 7, in the insert, r^2^ = 0.99) based on the Levenberg–Marquardt algorithm (OriginPro 8.5 SR1 software). By the analysis the association constants values calculated for dPro-HSA (K_a1_ = not achievable, n_1_ = not achievable, K_a2_ = 1.21 × 10^4^ ± 0.58 × 10^4^ mol·L^−1^, n_2_ = 0.95 ± 0.25) and dPro-oHSA (K_a1_ = 1.71 × 10^4^ ± 2.80 × 10^4^ mol·L^−1^, n_1_ = 0.49 ± 0.20, K_a2_ = 9.98 × 10^5^ ± 6.56 × 10^5^ mol·L^−1^, n_2_ = 0.70 ± 0.13) systems it can be concluded that the structure of HSA in IIIA subdomain (Sudlow’s site II) has been changed by the oxidation process, much more than the IIA subdomain. This phenomenon is consistent with the reports of Anraku et al. about the albumin changes by oxidation, mainly in Sudlow’s site II [14]. 

#### 2.2.2. 10*H*-3,6-Diazaphenothiazine (DAPT)-HSA and oHSA Binding

10*H*-3,6-diazaphenothiazine (DAPT) is a newly synthesized heterorganic compound which, due to its properties, may be used in the future in cancer therapy. The study of DAPT binding in terms of its binding to albumin, both oxidized (oHSA) and unmodified (HSA), is an innovative aspect of the study and may be the basis for further research on its introduction to the pharmaceutical market. The study of DAPT binding to albumin with the use of absorption and emission phenomena turned out to be relatively easy to measure. It is known that the fluorescence ability of the protein is mainly due to tryptophanyl and tyrosyl residues. Study of albumin fluorescence quenching provides information on drug binding. By observing the decrease in albumin fluorescence in the presence of ligand, the ligand binding to protein can be concluded [34].

Due to the fact that DAPT is an unknown in terms of albumin binding substance the possibility of energy transfer between protein fluorophores and aromatic ring of drug is obligatory to analyze and the absorption spectrum of DAPT and the emission spectrum of unmodified (HSA) and oxidized (oHSA) albumin (data not shown) were recorded. It is worth noting that the obtained spectra overlap. This phenomenon proves the short distance between the excited protein fluorophores, that is, a tryptophanyl and/or tyrosyl residues and an aromatic ring of DAPT molecule and thus energy transfer is possible. Stryer has reported that energy transfer, which can be observed as quenching of protein fluorescence, is possible only when the distance between the protein fluorophore and the quencher (drug) is sufficiently small and should not exceed 10 nm [34]. As a next part of the experiment emission fluorescent spectra of both HSA and oHSA in the presence of DAPT have been measured. The use of λ_ex_ 275 nm and λ_ex_ 295 nm excitation wavelengths allowed for the observation of tyrosyl or/and tryptophanyl fluorophores. These types of fluorophores are located in hydrophobic albumin subdomains, IIA for Trp-214 and Tyr-263, IIIA and IB for tyrosyl residues [48]. A decrease in albumin fluorescence intensity with the increase of DAPT concentration confirms the albumin fluorescence quenching as a result of absorption fluorophores energy by the ligand, regardless of oxidation. 

**Figure 8:**
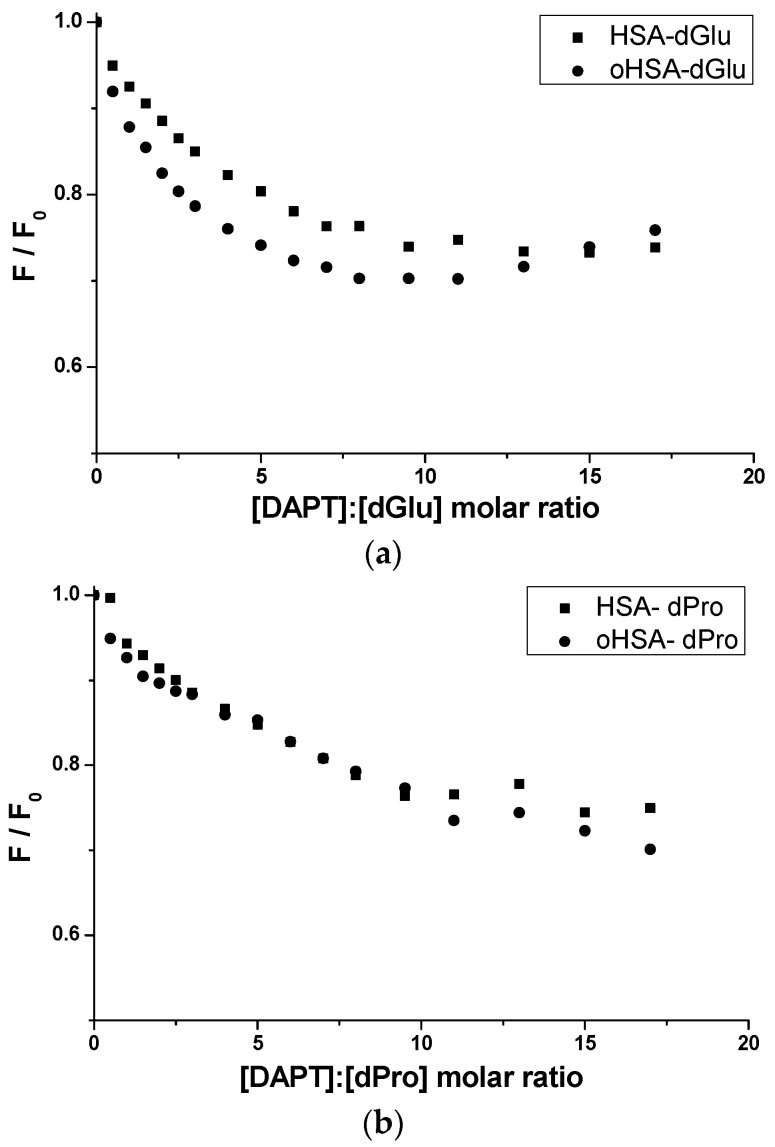
Comparison of quenching curves of (**a**) dGlu and (**b**) dPro at 5 × 10^−6^ mol·L^−1^ concentration in the presence of HSA, oHSA at 5 × 10^−6^ mol·L^−1^ concentration and DAPT (2.5 × 10^−6^ mol·L^−1^–8.5 × 10^−5^ mol·L^−1^); the error calculated as maximum deviation does not exceed the symbols.

To confirm the location of DAPT in HSA and oHSA molecules, one portion of dGlu/dPro fluorescent marker at 5 × 10^−6^ mol·L^−1^ concentration was added to albumin at 5 × 10^−6^ mol·L^−1^ concentration and next the systems dGlu-HSA, dGlu-oHSA, dPro-HSA, dPro-oHSA were titrated by DAPT (2.5 × 10^−6^ mol·L^−1^–8.5 × 10^−5^ mol·L^−1^). The fluorescence of the markers in each of the samples was measured (λ_ex_ 350 nm) (data not shown). Figure 8 presents a comparison of quenching curves of dGlu and dPro in the presence of HSA, oHSA and DAPT. 

Approximately 25% and 30% quenching of dGlu and dPro fluorescence by 10*H*-3,6-diazophenothiazine, respectively, was noted (Figure 8) and it probably means that ligand influences on the probes-albumin binding sites in subdomain IIA (dansyl-L-glutamine-albumin) and IIIA (dansyl-L-proline-albumin). Slightly higher quenching of dPro fluorescence by ligand (Figure 8b) proves that 10*H*-3,6-diazophenothiazine has better access to the IIIA subdomain by affecting one of the two dPro binding sites. Because dGlu binds to subdomain IIA with high association constant in one class of binding sites it can be a barrier for 10*H*-3,6-diazophenothiazine. However, the possibility of ligand location there was recorded, which is confirmed by the above-mentioned changes in the intensity of the fluorescence marker. Moreover, a slightly higher displacement of the marker from the oxidized protein was observed, which confirms the theory that the oxidation changed the structure of the macromolecule.

The quenching of fluorescence is manifested by a reduction in spectrum intensity. It is worth noting that various mechanisms underlie fluorescence quenching like static and dynamic quenching. Static quenching accompanies the formation of non-fluorescent complexes; however, this phenomenon can be dynamic when the activity of the fluorophore is reduced due to contact with another molecule (quencher) presented in the solution. The dynamic quenching is described by the Stern-Volmer equation [49]. If the Stern-Volmer plot has a straight course, the quenching can be static or dynamic and the quencher has the same access to all the same class fluorophores presented in molecule. In the case of a positive deviation from the rectilinear course of the Stern-Volmer plot, the quenching character is mixed—static and dynamic. If in the molecule there are two different populations of fluorophores with different quencher access, a negative deviation from the straight-line plot is observed [49]. Based on the data from the emission fluorescence spectra of both unmodified (HSA) and oxidized (oHSA) serum albumin in the presence of DAPT (data not shown), the Stern-Volmer plots have been drawn. 

**Figure 9:**
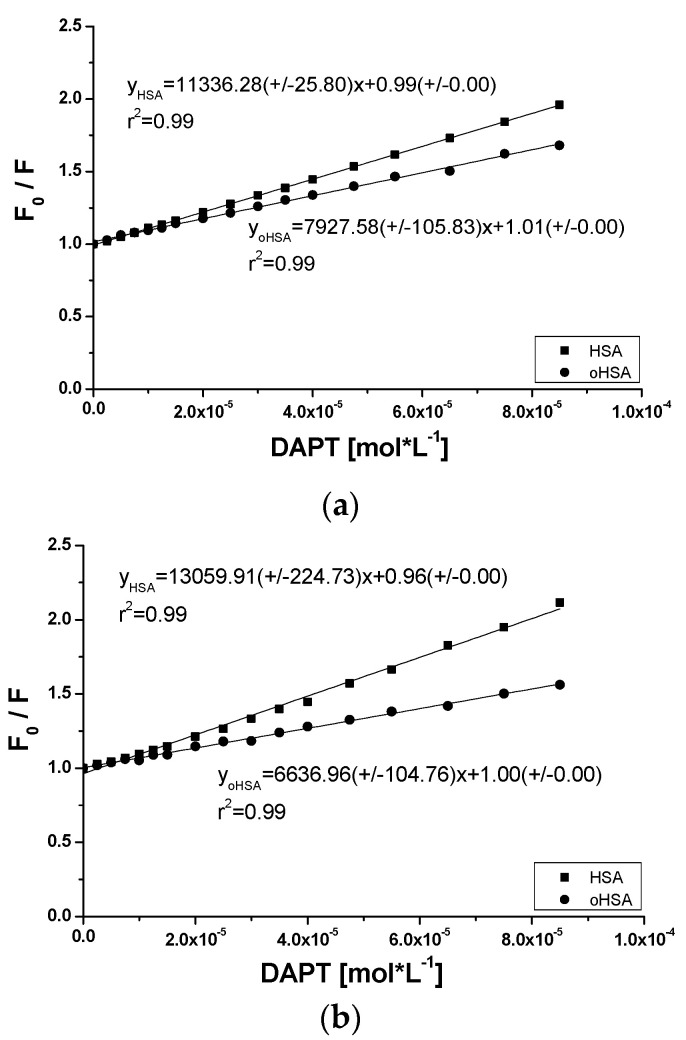
The Stern-Volmer curves of F_0_/F vs. 10*H*-3,6-diazophenothiazine (DAPT) concentration at (**a**) λ_ex_ 275 nm and (**b**) λ_ex_ 295 nm; the error calculated as maximum deviation does not exceed the symbols.

The straight line course of Stern-Volmer plots presented in Figure 9 indicates a dynamic or static quenching in the subdomain where fluorophores, taking part in the interaction [50] are located and the Stern-Volmer constants KSV have been calculated and equal to 1.13 × 104 mol·L^−1^ and 1.30 × 104 mol·L^−1^ in DAPT-HSA complex as well as in the complex of DAPT with oHSA equal to 0.79 × 10^4^ mol·L^−1^ and 0.66 × 10^4^ mol·L^−1^, at λ_ex_ 275 nm and 295 nm, respectively. As observed, the highest K_SV_ value was calculated for the ligand-HSA system excited at λ_ex_ 295 nm. In turn, the lowest value has been obtained for the ligand-oHSA system excited also at λ_ex_ 295 nm. The Stern-Volmer constant K_SV_ informs us about the availability of the quencher to the excited fluorophore and its increase points to the reduction of the distance between the ligand and macromolecule [51]. Based on Equation (5), bimolecular quenching rate constants k_q_ in mol^−1^Ls^−1^ have been calculated and are equal to 1.88 × 10^12^ mol^−1^Ls^−1^ and 2.17 × 10^12^ mol^−1^Ls^−1^ in DAPT-HSA complex as well as in the complex of DAPT with oHSA equal to 1.32 mol^−1^Ls^−1^ and 1.10 mol^−1^Ls^−1^, at λ_ex_ 275 nm and 295 nm, respectively. Lakowicz found that the maximum value of the constant k_q_ equal to 1 × 10^10^ mol^−1^Ls^−1^ concerns dynamic quenching of fluorescence in an aqueous solution, while above 1 × 10^10^ mol^−1^Ls^−1^ this value applies to static quenching [52]. Static quenching taking part in the interaction reduces the intensity of emitted albumin fluorescence at the time when the ligand (DAPT) in its un-excited state interacts with fluorophore molecules with the decrease of available and excitable fluorophores population. 

**Figure 10:**
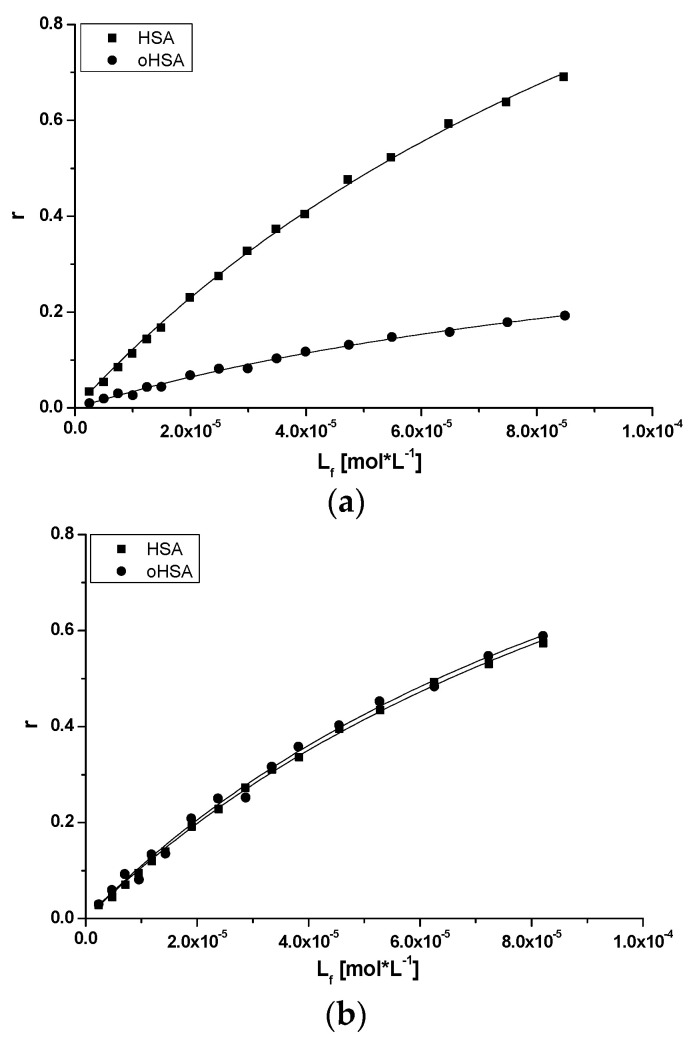
The binding isotherms for DAPT-HSA and DAPT-oHSA systems at (**a**) λ_ex_ 275 nm and (**b**) λ_ex_ 295 nm.

**Figure 11:**
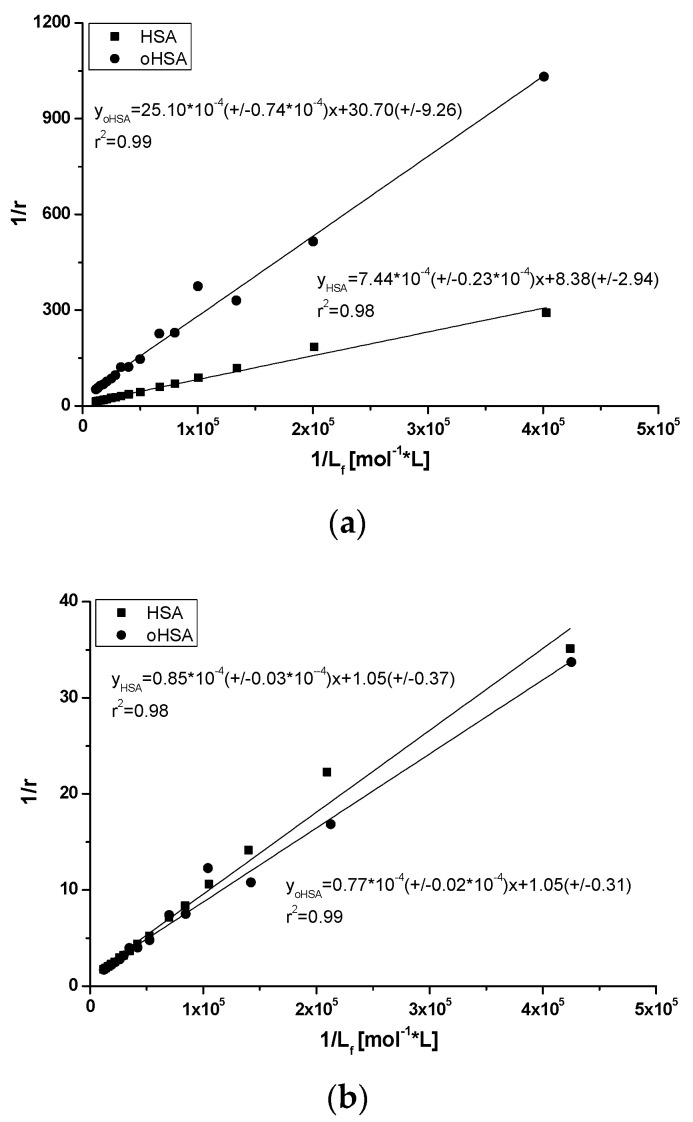
The Klotz plots for DAPT-HSA and DAPT-oHSA systems at (**a**) λ_ex_ 275 nm and (**b**) λ_ex_ 295 nm.

Using the data from emission fluorescence spectra of both HSA and oHSA in the presence of DAPT at λ_ex_ 275 nm and 295 nm, based on Equation (6), the Scatchard curves have been plotted. The Scatchard plots obtained for DAPT systems with HSA and oHSA are not linear (data not shown). This phenomenon means that there are more than one binding site for DAPT in subdomains IIIA and/or IIA of both oxidized oHSA and unmodified HSA. Moreover, it should be noted that subdomain IIA was divided by Yamasaki [10,53] into three regions: Ia, Ib and Ic, and DAPT can probably bind to one of these regions. Based on the binding isotherms (Equation (7), Figure 10) using the non-linear regression method (Levenberg–Marquardt algorithm using the computer program OriginPro 8.5 SR1, Northampton, MA, USA) the association constants K_a_ [mol^−1^·L] for two classes of binding sites were determined. Based on Equations (8) and (9), and the Klotz (Figure 11), curves have been plotted and K_a_, n values have been calculated. All calculated binding parameters have been collected in Table 5. 

Binding isotherms drawn based on Equation (7) allowed the evaluation of ligand-protein binding specificity. The course of the isotherms presented on Figure 10 exponentially grows, without reaching the *plateau*, which indicates both a nonspecific interaction with the hydrophobic surface fragments of unmodified and oxidized albumin and specific in the binding sites. 

Figure 11 presents the Klotz plots at the excitation wavelength λ_ex_ 275 nm and 295 nm. K_a_ values have been determined in Table 5. 

From the data collected in Table 5, an increase in the K_a_ constants was observed in the oxidized protein compared to the unmodified one. The number of ligand molecules n bound to one albumin molecule at excitation λ_ex_ 295 nm in both systems is the same. After excitation at λ_ex_ 275 nm the determination of n value was impossible. The data collected in Table 5 show that at excitation λ_ex_ 295 nm, an increase in K_a_ constants for the oxidized protein compared to the unmodified one was observed, but at excitation λ_ex_ 275 nm the decrease was registered. The ligand-oHSA system excited at λ_ex_ 295 nm shows the highest K_a_ constant value. 

The obtained values of K_a_, K_SV_ and n allow us to suppose that the complexes of the tested DAPT are more stable with the oxidized carrier than with the unmodified one. However, it should be noted that when comparing the values of the association constant and the number of n molecules, no significant differences can be seen between the DAPT—unmodified and oxidized albumin complexes. Probably, 10*H*-3,6-diazophenothiazine binds to subdomain IIIA and/or IIA, IB with the same strength and oxidation has only a slight influence on this binding. As a result, albumin oxidation should not affect its distribution in the body.

## 3. Materials and Methods

### 3.1. Chemicals

Human serum albumin, fraction V, Lot No 4971K (HSA) and dansyl-l-glutamine (dGlu) were purchased from MP Biomedicals, Inc. (Illkirch, France). Dansyl-l-proline (dPro) was obtained from Fluka Chemie AG (Buchs, Switzerland), methanol absolute Uvasol® for spectroscopy from Merck KGaA (Darmstadt, Germany). Chloramine T (ChT), sodium thiosulfate anhydrous (Na_2_S_2_O_3_), edetate disodium dehydrate (EDTA) were gained from CHEMPUR Poland (Piekary Śląskie, Poland) and 5.5′-Dithio-bis(2-nitrobenzoic acid) (DTNB), dimethyl sulfoxide (DMSO) from SIGMA-ALDRICH Chemie GmbH, (St. Louis, MO, USA). 10*H*-3,6-diazaphenothiazine (DAPT) was synthesized in the Department of Organic Chemistry, Faculty of Pharmaceutical Sciences in Sosnowiec, Medical University of Silesia in Katowice, Poland, according to described procedure [19]. 

### 3.2. Methods

#### 3.2.1. Human Serum Albumin Oxidation

Sample Preparation: Two albumin solutions at 4 × 10^−5^ mol·L^−1^, with (oxidized oHSA) and without (unmodified HSA) chloramine T (ChT) at 1 × 10^−3^ mol·L^−1^ concentration (ChT:HSA = ChT:oHSA 25:1) were incubated in 0.05 mol·L^−1^ phosphate buffer at pH 7.4 containing edetate disodium dehydrate (EDTA) (1 × 10^−3^ mol·L^−1^) for 60 min at 37 °C. After 60 min of incubation to oxidized HSA and unmodified HSA solutions at room temperature a sodium thiosulfate (Na_2_S_2_O_3_) at 3.6 × 10^−3^ mol·L^−1^ concentration (Na_2_S_2_O_3_:HSA = Na_2_S_2_O_3_:oHSA 90:1) and an appropriate volume of 0.05 mol·L^−1^ phosphate buffer, respectively, have been added. In order to estimate the degree of oxidation, samples of (1) DTNB (1.5 × 10^−4^ mol·L^−1^) in the presence of HSA (4 × 10^−5^ mol·L^−1^), (2) DTNB (1.5 × 10^−4^ mol·L^−1^) in the presence of oxidized HSA (4 × 10^−5^ mol·L^−1^), (3) HSA (4 × 10^−5^ mol·L^−1^) and (4) oxidized HSA (4 × 10^−5^ mol·L^−1^) in 0.05 mol·L^−1^ phosphate buffer at pH 7.4 containing EDTA (1 × 10^−3^ mol·L^−1^) have been prepared. A stock solution of ChT at 0.1 mol·L^−1^, sodium thiosulfate at 0.3 mol·L^−1^ and DTNB at 0.01 mol·L^−1^ concentrations were prepared in 0.05 mol·L^−1^ phosphate buffer, pH 7.4. 

Spectra Measurements: Emission and excitation fluorescence spectra of unmodified HSA and oxidized oHSA albumin were taken using spectrofluorimeter JASCO FP-6500 with quartz cuvette at 10 mm path length, T = 20 °C, parameters for emission spectra of unmodified HSA and oxidized HSA were: λ_ex_ 275 nm and λ_ex_ 295 nm at range 285–400 nm, 305–400 nm, respectively. Parameters for excitation spectra registered for HSA and oHSA: λ_ex_ 250–310 nm at λ_em_ 340 nm. In order to study “*Red Edge Excitation Shift*” effect of unmodified and oxidized albumin, HSA and oHSA solutions at 4 × 10^−5^ mol·L^−1^ were diluted to 5 × 10^−6^ mol·L^−1^ in 0.01 mol·L^−1^ phosphate buffer and excited at λ_ex_ 290 nm, λ_ex_ 295 nm, λ_ex_ 300 nm, λ_ex_ 305 nm (accuracy of wavelength is ± 1.5 nm). 

Absorption spectra of unmodified HSA and oxidized oHSA albumin were taken using spectrophotometer JASCO V-530 and the absorbance at 278 nm has been read. The degree of albumin oxidation (the molar concentration of free sulfhydryl groups) was estimated on the basis of absorbance measurements at the wavelength 412 nm (maximum absorption of Ellman’s reagent (DTNB)) of (1) DTNB in the presence of HSA, oHSA (Abs_412c_), and (2) HSA, oHSA (Abs_412p_) (DTNB:HSA = DTNB:oHSA 3:1 molar ratio) (Equation (1)) [11,54]. The albumin and DTNB concentrations were 4 × 10^−5^ mol·L^−1^ and 1.2 × 10^−4^ mol·L^−1^, respectively. The absorbance value of DTNB (Abs_412b_) was treated as background. To determine the percentage of free sulfhydryl groups [SH] in unmodified and oxidized albumin Equation (2), which determines the amount of free sulfhydryl groups per 100 molecules of albumin has been used [11,54].

(1) $$\left[\mathrm{SH}\right]=\;\frac{{\mathrm{Abs}}_{412c}\;-{\;\mathrm{Abs}}_{412b}\;-{\;\mathrm{Abs}}_{412p}}{{\Delta \varepsilon }_{412}\;\cdot \;1\mathrm{cm}},$$

where:

Δε_412_—molar absorption coefficient at 412 nm
Abs_412c_—absorbance of DTNB-HSA complex
Abs_412b_—absorbance of DTNB
Abs_412p_—absorbance of HSA

(2) $$\left[\mathrm{SH}\right]\%=\;\left[\mathrm{SH}\right]/\left[\mathrm{HSA}\right]\cdot 100\%=\frac{\left[\mathrm{SH}\right]}{\left[\mathrm{oHSA}\right]}\cdot 100\%,\;$$

where:

[SH]—molar concentration of free-SH groups in protein mol·L^−1^
[HSA] = [oHSA]—the molar concentration of albumin in mol·L^−1^.

Circular dichroism (CD) spectra of HSA and oHSA at 1 × 10^−6^ mol·L^−1^ concentration were recorded using JASCO J-1500 spectropolarimeter. The measurements were made at 20 °C, in quartz cuvettes with an optical path length 2 mm. The accuracy of the wavelength measurement was ±0.1 nm and the wavelength repeatability was ±0.05 nm. 

The mean residue ellipticity [θ]_MRW_:

(3) $${\left[\theta \right]}_{\;\mathrm{MRW}}=\frac{\mathrm{MRW}\cdot \theta }{10\cdot l\cdot m}\;\left[\deg\cdot {\mathrm{cm}}^{2}\cdot {\mathrm{dmol}}^{-1}\right],$$

where:

θ—observed ellipticity for a given wavelength in deg
m—concentration in g/cm^3^ [55,56]
l—optical path length in cm
MRW—mean residue weight, MRW_HSA_ = 113.7 Da.

Spectrum CD of buffer for unmodified albumin, spectrum CD of chloramine T and sodium thiosulfate or DTNB spectrum in buffer solution for oxidized albumin used to obtain the HSA and oHSA solutions was subtracted from each of the obtained spectra.

#### 3.2.2. Human Serum Albumin—Ligand Binding Assessment

Sample preparation: Each of HSA and oHSA solutions at 4 × 10^−5^ mol·L^−1^ concentration was diluted in 0.01 mol·L^−1^ phosphate buffer to 1 × 10^−5^ mol·L^−1^ and 5 × 10^−6^ mol·L^−1^. Using both HSA and oHSA at 1 × 10^−5^ mol·L^−1^ concentration, fluorescent probes dansyl-l-glutamine (dGlu) (5 × 10^−3^ mol·L^−1^) and dansyl-l-proline (dPro) (5 × 10^−3^ mol·L^−1^) binding with HSA and oHSA ([dGlu] = [dPro] 0.5 × 10^−5^ mol·L^−1^–6 × 10^−5^ mol·L^−1^) have been studied. 

Solutions of HSA and oHSA at 5 × 10^−6^ mol·L^−1^ concentration have been divided into two parts. From the first part, both HSA and oHSA solutions (5 × 10^−6^ mol·L^−1^) were titrated by DAPT (2.5 × 10^−6^ mol·L^−1^–8.5 × 10^−6^ mol·L^−1^), λ_ex_ 275 nm and λ_ex_ 295 nm. To the second part of HSA (5 × 10^−6^ mol·L^−1^) and oHSA (5 × 10^−6^ mol·L^−1^), dGlu (5 × 10^−6^ mol·L^−1^) and dPro (5 × 10^−6^ mol·L^−1^) fluorescent probes were added at HSA:dGlu = HSA:dPro = oHSA:dGlu = oHSA:dPro 1:1 molar ratios and the solutions were titrated by DAPT (2.5 × 10^−6^ mol·L^−1^–8.5 × 10^−5^ mol·L^−1^), λ_ex_ 350 nm. Stock solutions of dGlu and dPro at 2.5 × 10^−3^ mol·L^−1^ concentration were prepared in methanol while a stock solution of DAPT (2.5 × 10^−3^ mol·L^−1^) was prepared in dimethyl sulfoxide (DMSO).

Spectra Measurements: Using spectrofluorimeter JASCO FP-6500 emission fluorescent spectra of fluorescent probes dansyl-l-glutamine (dGlu), dansyl-l-proline (dPro) and HSA and oHSA titrated dGlu and dPro have been obtained, where λ_ex_ was 350 nm. In addition, HSA and oHSA solutions in the system with DAPT, without (DAPT-HSA, DAPT-oHSA) and in the presence of fluorescent probes (DAPT-dPro-HSA, DAPT-dPro-oHSA, DAPT-dGlu-HSA, DAPT-dGlu-oHSA) have been studied, λ_ex_ 275 nm, λ_ex_ 295 nm. 

Due to the absorption of light at both excitation and emission wavelengths (inner filter effect, IFE), a correction of DAPT-albumin systems fluorescence intensity is required. The absorbance measurements at the wavelength used to excite fluorophores fluorescence and at emission wavelength were made using a JASCO V-530 spectrophotometer and for the inner filter correction Equation (4) has been used [52].

(4) $$F_{\mathrm{cor}}=F_{\mathrm{obs}}\cdot 10^{\frac{\mathrm{Aex}+\mathrm{Aem}}{2}}\;\;,$$

where:

F_cor_ and F_obs_—corrected and observed fluorescence (after subtraction the scattering spectrum of solvent), respectively
A_ex_ and A_em_—the absorbance at the excitation and emission wavelength, respectively.

The kinetics of the human serum albumin interaction with fluorescence quenching substance is represented by the Stern-Volmer equation [49]:

(5) $$\frac{F_{0}}{F}=1+k_{q}\tau_{0}\left[Q\right]=1+K_{\mathrm{SV}\;}\left[Q\right],$$

where:

F, F_0_—fluorescence intensity at the maximum wavelength of albumin in the presence and absence of a quencher, respectively
k_q_—bimolecular quenching rate constant in mol·L^−1^·s^−1^
τ_0_—the average fluorescence lifetime of HSA without of quencher, τ_0_ = 6.0 × 10^−9^ s [57]
[Q]—ligand concentration in mol·L^−1^
K_SV_—Stern-Volmer constant in mol·L^−1^.

The association constants K_a_ in ligand-protein systems has been determined by the Scatchard [58] (Equation (6)), binding isotherms [59] (Equation (7)), Klotz [60] (Equation (8)).

(6) $$\frac{r}{L_{f}}={\mathrm{nK}}_{a}-K_{a}\cdot r.\;$$

Scatchard equation: 

where:

n—number of binding sites classes
r—number of ligand moles bound to 1 mole of protein; $r=\frac{L_{b}}{\left[\mathrm{HSA}\right]}$
L_b_—bound ligand concentration in mol·L^−1^
[HSA]—total protein concentration in mol·L^−1^
L_f_—free ligand concentration in mol·L^−1^
K_a_—association constant in mol·L^−1^.

When the Scatchard curve is not straight, this indicates the presence of more than one class of binding site. For two classes of binding sites in HSA structure, according to Equation (7) the binding isotherms using non-linear regression analysis were drawn and the association constants K_a1_ and K_a2_ and the number of binding sites n_1_, n_2_ were calculated:

Binding isotherm:

(7) $$r=\frac{n_{1}\cdot K_{a1}\cdot \left[L_{f}\right]}{1+K_{a1}\cdot \left[L_{f}\right]}+\frac{n_{2}\cdot K_{a2}\cdot \left[L_{f}\right]}{1+K_{a2}\cdot \left[L_{f}\right]},$$

where

n_1_, n_2_—are the numbers of binding sites
K_a1_, K_a2_—the association constants in mol·L^−1^.

Klotz equation:

(8) $$\frac{1}{r}=\frac{1}{n}+\frac{1}{n\cdot K_{a}\cdot L_{f}}.\;$$

### 3.3. Statistics 

The results of the study were expressed as a mean ± relative standard deviation (SD) from three independent experiments. Linear regression was analyzed using OriginPro version 8.5 SR1 software (Northampton, MA, USA) by fitting experimental data to the corresponding equation.

## 4. Conclusions

The knowledge obtained from the analysis of potential therapeutic substance binding with its carrier in the blood is one of the time-consuming and costly steps allowing for its subsequent introduction to the pharmaceutical market. Such studies also aim to develop a model for the enhancement of the therapeutic effect of a drug and to assess adverse side effects. Moreover, the awareness of the presence of medicinal substances carrier modifications allows for understanding serious and constantly emerging diseases. The conducted research carried out for HSA and oHSA and their systems with 10*H*-3,6-diazaphenothiazine (DAPT)—newly synthesized substance, allowed us to obtain promising and innovative conclusions about the pharmacokinetic aspects of 10*H*-3,6-diazaphenothiazine and its biological properties. DAPT has strong an anticancer activity and, so far, no studies have been conducted to analyze its interaction with the transporting protein—human serum albumin, both oxidized (oHSA) and unmodified (HSA), which makes our research fundamental. Moreover, in order to mimic physiological conditions and the possibility of HSA modification under the influence of oxidation stress during cancer therapy, HSA molecule has been incubated (oxidized) with chloramine T for 60 minutes at pH 7.4 and T = 37 °C. The results obtained using spectroscopic techniques showed conformational changes in the structure of the albumin molecule. Changes in the conformation of the oHSA molecule can probably affect its ability to bind ligands because the surroundings of Sudlow’s sites, in particular site II, have become more hydrophobic and rigid. Based on the obtained data we concluded that DAPT binds, regardless of its modification with chloramine T, specifically in Sudlow’s binding site I and II, which has been confirmed by the quenching of dansylated amino acids, dansyl-l-glutamine (dGlu) and dansyl-l-proline (dPro). Although the stronger effect of chloramine in the oxidation of Sudlow’s site II has been indicated, it is proved that this does not affect the formation of the complexes. It can be concluded that, regardless of the commonly occurring oxidative stress, plasma protein can probably be a carrier of DAPT and cancer monitoring therapy should be taken into account. Moreover, the obtained fundamental results could contribute to assess the diazaphenothiazine mechanism action in the organism and taken together with other data, such as the analysis of nanoparticles as ligand carriers, can be useful in the design of anticancer therapeutics. Due to the promising results from the molecular research point of view, as a next part of this paper, DAPT binding with alpha 1-acid glycoprotein (AGP) is planning to study. 

## Figures and Tables

**Table 1:** Fluorescence of unmodified (HSA) and oxidized (oHSA) human serum albumin at 5 × 10^−6^ mol·L^−1^ concentration; the excitation wavelengths λ_ex_ 275 nm and λ_ex_ 295 nm.

SA	λ_ex_ 275 nm				λ_ex_ 295 nm			
	λ_max_ [nm]	F_max_	FWHM [nm]	A	λ_max_ [nm]	F_max_	FWHM [nm]	A
HSA	333	189.67	59.32	0.77	338	66.25	54.64	1.10
oHSA	328	47.11	56.84	0.41	337	53.13	52.55	1.05

**Table 2:** Fluorescence of unmodified (HSA) and oxidized (oHSA) human serum albumin at 5 × 10^−6^ mol·L^−1^ concentration; the excitation wavelengths λ_ex_ 290 nm, λ_ex_ 295 nm, λ_ex_ 300 nm, λ_ex_ 305 nm.

SA	λ_ex_ 290 [nm]	λ_ex_ 295 [nm]	λ_ex_ 300 [nm]	λ_ex_ 305 [nm]	∆λ_max_ [nm]
	λ_max_ [nm]				
HSA	338	338	336	341	3
oHSA	334	337	339	346	12

**Table 3:** [θ_MRW_] at λ_min_ for 1 × 10^−6^ mol·L^−1^ HSA and oHSA.

SA	λ_min_ [nm]	θ_MRW_ [deg·cm^2^·dmol^−1^]	λ_min_ [nm]	θ_MRW_ [deg·cm^2^·dmol^−1^]
HSA	209	−25,054.60	223	−21,145.30
oHSA	209	−22,558.70	223	−19,489.20

**Table 4:** The percentage (%) content of the secondary structure elements of HSA and oHSA. Yang’s reference model.

SA	% α-Helix	% β-Sheet	% Other (Turn + Random)
HSA	27.6	11.0	61.4
oHSA	24.3	19.3	56.4

**Table 5:** Binding parameters for DAPT-HSA and DAPT-oHSA systems at λ_ex_ 275 nm and λ_ex_ 295 nm.

	λ_ex_ 275 nm		λ_ex_ 295 nm	
	K_a_ × 10^4^ ± SD [mol^−1^·L]	n ± SD	K_a_ × 10^4^ ± SD [mol^−1^·L]	n ± SD
DAPT-HSA	1.12	N/A*	1.23	0.95
DAPT-oHSA	1.22	N/A*	1.36	0.95

*N/A—not achievable.

## Data Availability

Not applicable.
